# A novel upper-limb function measure derived from finger-worn sensor data collected in a free-living setting

**DOI:** 10.1371/journal.pone.0212484

**Published:** 2019-03-20

**Authors:** Sunghoon Ivan Lee, Xin Liu, Smita Rajan, Nathan Ramasarma, Eun Kyoung Choe, Paolo Bonato

**Affiliations:** 1 College of Information and Computer Sciences, University of Massachusetts, Amherst, MA, United States of America; 2 ArcSecond Inc., San Diego, CA, United States of America; 3 College of Information Studies, University of Maryland, College Park, MD, United States of America; 4 Department of Physical Medicine and Rehabilitation, Harvard Medical School, Spaulding Rehabilitation Hospital, Charlestown, MA, United States of America; Washington University in Saint Louis School of Medicine, UNITED STATES

## Abstract

The use of wrist-worn accelerometers has recently gained tremendous interest among researchers and clinicians as an objective tool to quantify real-world use of the upper limbs during the performance of activities of daily living (ADLs). However, wrist-worn accelerometers have shown a number of limitations that hinder their adoption in the clinic. Among others, the inability of wrist-worn accelerometers to capture hand and finger movements is particularly relevant to monitoring the performance of ADLs. This study investigates the use of finger-worn accelerometers to capture both gross arm and fine hand movements for the assessment of real-world upper-limb use. A system of finger-worn accelerometers was utilized to monitor eighteen neurologically intact young adults while performing nine motor tasks in a laboratory setting. The system was also used to monitor eighteen subjects during the day time of a day in a free-living setting. A novel measure of real-world upper-limb function—comparing the duration of activities of the two limbs—was derived to identify which upper limb subjects predominantly used to perform ADLs. Two validated handedness self-reports, namely the Waterloo Handedness Questionnaire and the Fazio Laterality Inventory, were collected to assess convergent validity. The analysis of the data recorded in the laboratory showed that the proposed measure of upper-limb function is suitable to accurately detect unilateral vs. bilateral use of the upper limbs, including both gross arm movements and fine hand movements. When applied to recordings collected in a free-living setting, the proposed measure showed high correlation with self-reported handedness indices (i.e., *ρ* = 0.78 with the Waterloo Handedness Questionnaire scores and *ρ* = 0.77 with the Fazio Laterality Inventory scores). The results herein presented establish face and convergent validity of the proposed measure of real-world upper-limb function derived using data collected by means of finger-worn accelerometers.

## Introduction

Upper-limb impairments can significantly limit the ability of individuals to perform essential activities of daily living (ADLs) and negatively impact their quality of life [1–4]. Monitoring real-world upper-limb function in individuals with upper-limb impairments, especially for those undergoing rehabilitation after a neurological event such as a stroke [5, 6], can provide clinically important information. About 75% of the individuals who survive a stroke—one of the leading causes of disability in adults [7]—experience long-term motor impairments of the stroke-affected upper-limb that limit their ability to perform ADLs [8]. Whilst an important goal of rehabilitation interventions in this patient population is to improve motor function of the stroke-affected upper-limb, scientific studies have shown that functional improvements achieved in the clinic do not always translate into an improvement in the amount and quality of upper-limb use in the home and community settings [9]. In other words, stroke survivors may show improvements in their motor abilities (i.e., *what they are capable of doing*) in the clinic that do not result in an increased use of their stroke-affected limb during the performance of ADLs outside of the clinic (i.e., *what they actually do*) [9, 10]. This is important because the lack of use of the stroke-affected limb leads to a phenomenon referred to as *learned non-use* [6, 11] that negatively affects brain plasticity phenomena that are key to maximizing the recovery of motor functions. Therefore, an objective assessment of upper-limb use during the performance of real-world ADLs is of paramount importance as it allows clinicians to estimate the real-world impact of rehabilitation interventions, and it can enable self-management and patient-driven therapy [12].

Over the past decade, wrist-worn accelerometers have gained tremendous interest among researchers and clinicians as a low-cost, objective tool to quantify real-world upper-limb function [12]. Measures of upper-limb function, derived from wrist-worn accelerometers—often placed bilaterally –, can be broadly categorized into: 1) *intensity of limb use* [9, 10, 12–19], 2) *time duration of limb use* [19–24], and 3) *use ratio of the two limbs* [14, 15, 19, 21, 22, 25–28]. The *intensity of limb use* is computed by integrating the acceleration magnitude time-series derived from the accelerometer data collected during the monitoring period. Several studies have investigated the intensity of unilateral (e.g., stroke-affected side only) as well as bilateral (i.e, both limbs combined) upper-limb activities. The *time duration of limb use* is a measure of the amount of time during which subjects actively use the stroke-affected limb. Active vs. inactive periods are determined by estimating the time intervals during which the acceleration magnitude exceeds a set threshold value. The *use ratio of the two limbs* measures the activity of the stroke-affected limb and compares it to the activity of the contralateral limb. The *use ratio of the two limbs* can be computed for the *intensity of limb use* as well as the *time duration of limb use*.

Despite the growing interest for their potential application as a tool to monitor real-world upper-limb movements, wrist-worn accelerometers have shown a number of limitations that hinder their widespread adoption in the clinic [12]. Wrist-worn accelerometers are capable of capturing arm and forearm movements (i.e., gross arm movements) but they do not allow one to accurately monitor movements of the hand and fingers (i.e., fine hand movements), which are of great relevance to monitoring ADLs [12, 17]. Consequently, measures derived from wrist-worn accelerometers have been criticized for underestimating movement intensity and their inability to precisely capture changes in the performance of upper-limb movements that are relevant to the assessment of motor abilities [29]. Furthermore, previous studies relying on measures of upper-limb use derived from wrist-worn accelerometers have not shown significant differences in the use of the dominant vs. the non-dominant limb during the performance of real-world ADLs in neurologically intact individuals [12, 18, 22], despite evidence supporting that subjects indeed use their dominant limb more frequently [30]. We hypothesize that this is because wrist-worn accelerometers only capture gross arm movements, but they do not yield accurate information related to fine hand movements that are of paramount importance in the context of monitoring goal-directed use of the upper limbs.

Previous studies have pursued the development and assessment of various technologies to monitor fine hand movements. Several researchers have developed and tested gloves with embedded sensors. For instance, piezoresistive fabric [31] and inertial sensors mounted on flexible substrates [32] have been knitted on or attached to the dorsal side of gloves to monitor fine hand movements. Unfortunately, wearing a glove negatively affects tactile feedback, which is key to accomplishing a number of ADLs. Others have relied on collecting electromyographic data from extrinsic muscles as a proxy measure of fine hand movements [33, 34]. However, collecting accurate electromyographic data in free-living conditions is challenging, because it is difficult to maintain a stable skin-electrode contact over long periods of time. Besides, data from extrinsic muscles provide only an indirect measure of fine hand movements. Direct measures of hand and finger movements are more desirable. To gather direct measures of fine hand movements, Friedman *et al*. [35] proposed to use a magnetic ring worn on the index finger and two triaxial magnetometers mounted in a watch-like unit. Unfortunately, this technology is negatively affected when subjects are in the proximity of or manipulate ferromagnetic materials, which is a common instance in everyday life.

In this paper, we investigate the use of finger-worn accelerometers to monitor gross arm and fine hand movements. This technology provides direct measures of upper-limb movements and is not affected by the proximity of ferromagnetic materials. Hence, it is ideally suited to quantify upper-limb function (i.e., herein intended as activity) [36–39] in real-world conditions. Furthermore, we introduce a novel measure of upper-limb function based on comparing the time duration of the (sufficiently intensive) use of the two upper limbs (e.g., dominant vs. non-dominant limb or stroke-affected vs. contralateral limb). Hence, the proposed measure quantifies the difference in the amount of the two limbs during the performance of ADLs (i.e., *use ratio of the two limbs*). This study examines the validity of the proposed approach by collecting and analyzing data from neurologically intact individuals in a laboratory and a free-living environment as a preliminary step toward developing a system suitable to monitor stroke survivors in the home and community setting.

## Materials and methods

### Wearable sensors

**Fig 1:**
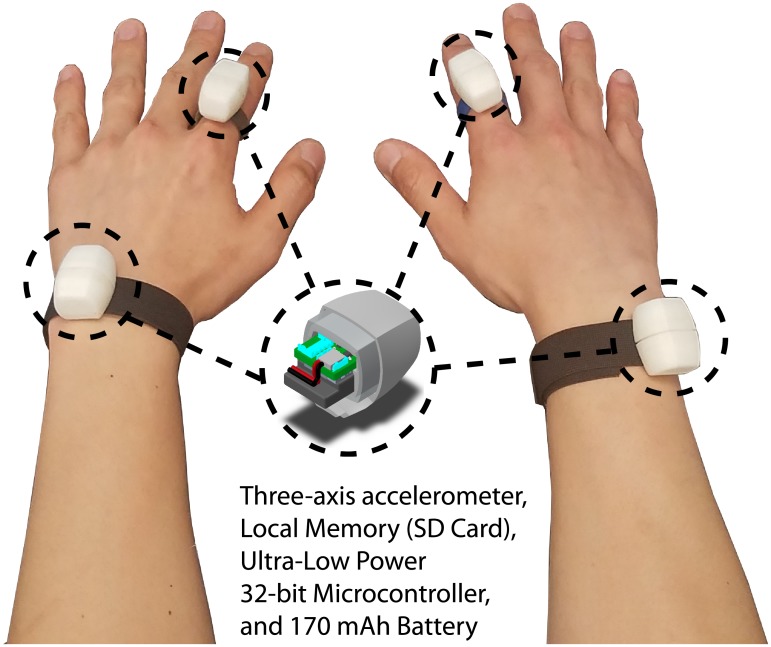
The miniaturized finger-worn and wrist-worn sensors used in the study. The sensor consists of a three-axis accelerometer enclosed in a waterproof case.

The miniaturized sensor that we used in the study (Arcus, ArcSecond Inc., USA) consisted of a three-axis accelerometer, a local memory for data storage (i.e., a micro-SD card), a 170 mAh battery, and an ultra-low-power 32-bit microprocessor in a waterproof enclosure (see Fig 1). We placed sensing components on the finger and the wrist using a ring and a wristband, respectively. In the study, the accelerometer data was sampled at 67 *Hz* and stored in the local memory to be retrieved upon completion of the data collection for offline analyses. We previously validated the sensor’s ability to quantify the amount of fine hand movements in a laboratory setting [36–38]. In this study, we focused on the suitability of the technology to derive a novel measure of upper-limb function based on comparing the amount of use of the two limbs during the performance of ADLs.

### Participants

A total of 35 neurologically-intact, able-bodied individuals were recruited by word of mouth from the University of Massachusetts Amherst to participate in two different sets of experiments: 1) in-laboratory and 2) free-living experiments. Eighteen subjects (21.7 ± 2.3 years old; 5 females) participated in the in-laboratory experiments and 18 subjects (23.4 ± 4.2 years old; 4 females) in the free-living experiments; one subject participated in both. Among the subjects who participated in the in-laboratory experiments, one was left-handed, one was ambidextrous, and the remaining subjects were right-handed. Among the subjects who participated in the free-living experiments, five subjects were left-handed and the remaining subjects were right-handed. Subjects were 18 to 40 years of age and had no history of orthopedic, musculoskeletal, neurological, or any other disorder or medical condition that could result in aberrant upper-limb movement patterns or affect subjects’ ability to perform the motor tasks independently. The experimental procedures were approved by the Institutional Review Board (IRB) of the University of Massachusetts Amherst (IRB# 2017-3628). All subjects provided written consent to participate in the study.

### In-laboratory experiments

The primary objective of the in-laboratory experiments was to determine the characteristics of data collected using the finger-worn accelerometer data during the performance of a set of motor tasks involving various limb-use intensity and laterality. This data allowed us to develop a measure of real-world upper-limb function that we later applied to the data collected in free-living conditions. Specifically, we used the data collected in the laboratory to investigate 1) the ratio of limb use, a measure to distinguish unilateral vs. bilateral tasks, and 2) limb-use intensity (i.e., acceleration magnitude) during the performance of motor tasks. When subjects arrived in the laboratory, we instructed them to wear the above-mentioned sensors on the wrists and index fingers, as shown in Fig 1. Although we had hypothesized that using the finger-worn sensors would have been sufficient to yield accurate information regarding upper-limb function, we also placed sensors on the wrists to enable a comparative analysis against previous studies that used wrist-worn sensors. Subjects were asked to perform nine different motor tasks listed in Table 1. A similar set of motor tasks was used in previous studies [17, 36, 40]. It is worth emphasizing that *limb laterality* in Table 1 is meant to highlight whether one upper limb or both upper limbs were used to perform a given task. Motor tasks in the *Bilateral* category involved the use of both limbs throughout the performance of the entire motor task. Similarly, *unilateral* motor tasks involved the use of only one limb during the performance of the task. Motor tasks in the *Unilateral & Bilateral* category involved a mixture of bilateral (e.g., typing keys using both hands) and unilateral (e.g., use the right hand to press the Enter or Backspace keys) movements. Subjects were instructed to clap their hands while straightening the wrists three times before and after the performance of each motor task. The acceleration peaks from the four sensors were used to manually synchronize the time clocks.

**Table 1:** Motor tasks—mimicking various types of real-world ADLs—that subjects performed in a controlled laboratory setting.

Task	Description	Limb Laterality
1	Walking	Bilateral
2	Buttoning a shirt	Bilateral
3	Tying shoelaces	Bilateral
4	Typing on a keyboard	Unilateral & Bilateral
5	Folding a towel	Unilateral & Bilateral
6	Cutting putty dough with a fork and a knife	Unilateral & Bilateral
7	Opening a screw-top jar	Unilateral & Bilateral
8	Taking the cap off of a bottle and drinking	Unilateral & Bilateral
9	Flipping pages of a magazine	Unilateral

### Free-living experiments

The free-living experiments were conducted approximately one year after the completion of the in-laboratory experiments. Once subjects arrived in the laboratory (typically around 8 am), they were evaluated to determine their handedness—defined as the upper limb that they preferred to use during the performance of ADLs—based on two validated self-reported handedness indices: the Waterloo Handedness Questionnaire [41–43] and the Fazio Laterality Inventory [44, 45].

These well-established self-reported indices of handedness were used to assess convergent validity of the proposed finger-worn accelerometer based measure of upper-limb function. The proposed approach is similar to previous studies that used the Amount of Use (AOU) scale of the Motor Activity Log (MAL)—a standardized clinical assessment tool meant to capture real-world upper-limb performance in stroke survivors—to validate accelerometer-based measures [15, 26, 46–48]. The MAL-AOU is a self-reported measure based on a six-point ordinal scale (0-5) to evaluate patients’ use of the limbs (i.e., stroke-affected vs. contralateral limb use) in terms of time duration (e.g., always, most of the time, rarely, etc.) for 30 different ADLs (e.g., brushing teeth, using a fork, buttoning a shirt, etc.) [49]. A score of 0 indicates that the subject did not use the stroke-affected limb at all, whereas a score of 5 indicates that the subject used the affected limb as often as before the stroke [50]. The average score over the 30 items of the scale is utilized as a measure of upper-limb use. This is fundamentally the same as what handedness indices aim to measure, i.e., use of the dominant vs. non-dominant limb during the performance of ADLs.

The Waterloo Handedness Questionnaire evaluates individuals’ overall use preference of the right vs. left upper limb. It is based on individuals’ self-report of upper-limb use preference. The self-report of time duration of use of the upper limbs relies on a five-point ordinal scale (-2 to +2) meant to capture upper-limb use during the performance of 36 ADLs. The values of the ordinal scale are meant to represent the following situations: always using the left hand (i.e., 95% or more of the times), usually using the left hand (i.e., between 75% and 95% of the times), equally using the two hands (i.e., use each hand about 50% of the times), usually using the right hand, and always using the right hand. Limb use preference is quantified by adding all the individual item scores. The Fazio Laterality Inventory evaluates individuals’ limb use preference in a similar manner. Namely, it is based on subjects’ self-report of the percentage of times (0% to 100%) they use their right limb to perform 100 different ADLs. The average percentage score is used to represent the overall limb use preference.

After completing the handedness tests, subjects were instructed to wear the sensors on their wrists and fingers bilaterally during the day time of a weekday (i.e., for a period of approximately six to eight hours) and go about their normal daily routines. Prior studies show that a wear time of a day is sufficient to assess the feasibility and validity of capturing real-world limb use by means of wearable sensors [12, 20, 22]. Subjects were also asked to perform the clapping action before they left the laboratory for manual clock synchronization among the four sensors. All subjects were university students and their activities reflected a wide range of ADLs including walking to classes, going to restaurants, socializing, and tasks performed at a desk (e.g., writing and typing). They were reminded every one hour by automatic text messages to self-annotate a summary of their activities during the previous hour. We used this data to assess the face validity of the proposed sensor-based measure. The reminder also contained a message to perform the clapping for time synchronization to minimize the clock drifts among the four sensors. Once subjects completed the experiment and returned to the laboratory, they were asked to clap their hands again to finalize the data collection. Then, the accelerometer data stored in the SD cards was manually transferred to a computer and time-synchronized for analysis.

### Objective quantification of real-world upper limb use

In this section, we discuss the analytic methods that we used to quantify real-world upper-limb function based on the accelerometer data obtained from the sensors. We focused on capturing the time duration of unilateral limb activities with sufficient intensity, aiming to determine which limb each subject used more frequently during the performance of real-world ADLs.

The gravity-free acceleration magnitude (i.e., activity intensity) time-series was derived from the sensor data as follows: $|a_{r}|=\sqrt{a_{r,x}^{2}+a_{r,y}^{2}+a_{r,z}^{2}}-g$ and $|a_{l}|=\sqrt{a_{l,x}^{2}+a_{l,y}^{2}+a_{l,z}^{2}}-g$ for the sensors positioned on the right and left limbs, respectively. In the above equations, *g* = 9.8 *m*/*s*^2^ represents gravity and the accelerometer data was expressed in units of *g*. A low-pass filter with a cut-off frequency of 8 *Hz* was applied to the acceleration magnitude time-series to attenuate potential high-frequency noise components. The acceleration magnitude time-series were then down-sampled by averaging the data over one-second epochs in order to represent the comprehensive intensity of limb use during the performance of ADLs (rather than considering the intensity of every data sample captured at 67 *Hz*). While various epoch sizes have been used in the literature—ranging from a fraction of a second to a few minutes [12, 51]—one-second epoch is the most widely accepted length for monitoring upper-limb performance [13, 17, 19, 22, 28]. Herein, the down-sampled accelerometer data for the right and left limbs are referred to as |*a*_*r*_[*t*]| and |*a*_*l*_[*t*]| respectively, where *t* = 1, 2, 3, ⋯ seconds.

A limb was considered active when the acceleration magnitude time-series exceeded a set threshold. The threshold value was set relative to observations gathered at rest, herein intended as when the sensor was placed stationary on a table. In such conditions, the mean and standard deviation of the acceleration magnitude time-series were 0.016*g* ± 0.0015*g*, respectively. Epochs with an average acceleration magnitude greater than the 99.5% confidence interval of the stationary profile (i.e., 0.016*g* + 2.57 × 0.0015*g* = 0.020*g*) were classified as epochs of active upper-limb movements. Otherwise, the epochs were classified as associated with no activity.

When movement was detected, the ratio of activity intensity between the two limbs was computed to assess whether a single limb (either left or right) or both limbs were used during each epoch. This approach is similar to that taken in previous studies to measure upper-limb activity [14, 15, 19, 21, 22, 25–28]. Accordingly, the ratio of activity intensity was defined as:

$$\begin{array}{c} r\left[t\right]=\text{ln}(\frac{|a_{r}\left[t\right]|}{|a_{l}\left[t\right]|})=\text{ln}\left(|a_{r}\left[t\right]|\right)-\text{ln}\left(|a_{l}\left[t\right]|\right). \end{array}$$ (1)

The natural log was applied to the ratio of |*a*_*r*_[*t*]|/|*a*_*l*_[*t*]| in order to generate equal values in magnitude but opposite in sign for right-hand vs. left-hand activities [15]. When the absolute value of *r*[*t*] was smaller than a set threshold *δ*, we considered the two limbs as equally active (i.e., bilateral limb use). On the other hand, when *r*[*t*] > *δ*, we considered the right limb as used predominantly, and when *r*[*t*] < −*δ* we considered the left limb as used predominantly. Then, real-world upper-limb performance was quantified as follows:

$$\begin{array}{c} M=\frac{\left|t\in \{r\left[t\right]>\delta ,|a_{r}\left[t\right]|>\beta \}\right|}{T}-\frac{\left|t\in \{r\left[t\right]<-\delta ,|a_{l}\left[t\right]|>\beta \}\right|}{T}, \end{array}$$ (2)

where |*t* ∈ {⋅}| is the number of one-second epochs that satisfied the condition in the bracket, and *T* is the total monitoring duration in seconds. *β* represents a parameter identifying upper-limb activities (epochs) with sufficient intensity to be counted towards our measurement *M*. Optimal values of *δ* and *β* were derived as discussed in the following subsection.

*M* captures the difference in time duration of the activities performed using the two limb. A positive value of *M* indicates that the subject spent more time using the right upper limb to perform ADLs. Similarly, a negative value of *M* indicates that the subject spent more time using the left limb. Values of *M* close to zero indicate that the subject used equally—on average—the two limbs throughout the day (e.g., used the right limb for certain tasks—for instance to brush their teeth—while they used the left limb for other tasks—for instance to open doors).

For a comparative analysis, the proposed measure *M* was derived independently from 1) the finger-worn sensors, 2) the wrist-worn sensors, and 3) the difference between the acceleration magnitude time-series derived from the finger-worn and the wrist-worn sensors (i.e., the acceleration magnitude time-series of the finger-worn sensor minus that of the wrist-worn sensor for each limb separately). As discussed above, we hypothesized that finger-worn sensors alone could provide sufficient information to monitor gross arm movements and fine hand movements. We derived the difference between the acceleration magnitude time-series collected using the finger-worn and the wrist-worn sensors as an attempt to estimate an acceleration time-series capturing solely the movements of the hand and fingers relative to the wrist.

We assessed convergent validity of the proposed measure *M* against the two handedness indices chosen for the study (i.e., the Waterloo Handedness Questionnaire index and the Fazio Laterality Inventory index) by performing a Pearson correlation analysis. The proposed measure of upper-limb function was also compared to previously studied accelerometer-based measures including 1) the median of the activity ratio (i.e., *r*[*t*]) of the two limbs [17, 19], 2) the ratio of active use duration of the two limbs [20–22, 24, 46], 3) the ratio (between the two limbs) of the sum of the maximum acceleration magnitudes within each one-second epoch [15], 4) the percentage of dominant limb active periods compared to the total monitoring duration [17, 21], and 5) the time duration of bilateral limb activity (i.e., |*a*_*r*_[*t*]| + |*a*_*l*_[*t*]|) [17].

### Optimization of the parameters of the algorithm

Both the datasets collected from the in-laboratory and free-living experiments were used to identify the optimal values of *δ* and *β* in(2). The in-laboratory sensor data were used to establish the search space (i.e., the value range in which we assume that the optimal values are located) of the two parameters. More specifically, the search range for an optimal *δ* value—the parameter to detect unilateral use of the limbs—included the mean values of *r*[*t*] for the motor tasks that involved both unilateral and bilateral use of the limbs, i.e., Tasks #4-8 in Table 1. The search range for an optimal *β* value—the parameter to detect limb activity with sufficient intensity—was set from 0.020*g* (i.e., the threshold for active arm use) to the average activity intensity of the motor task that only involved unilateral limb use, i.e., Task #9 in Table 1.

Based on this established 2D search space of *δ* and *β*, we linearly increased the values within the range (see the Results section for details) and computed the proposed measure of upper-limb performance *M* for each subject who participated in the free-living experiment. This resulted in different values of *M* for different value combinations of *δ* and *β* (i.e., *M* was considered as a function of *δ* and *β*). We empirically identified the optimal values of the parameters that maximized the Pearson correlation coefficients to the handedness indices.

## Results

### In-laboratory experiments—Performance of motor tasks

**Fig 2:**
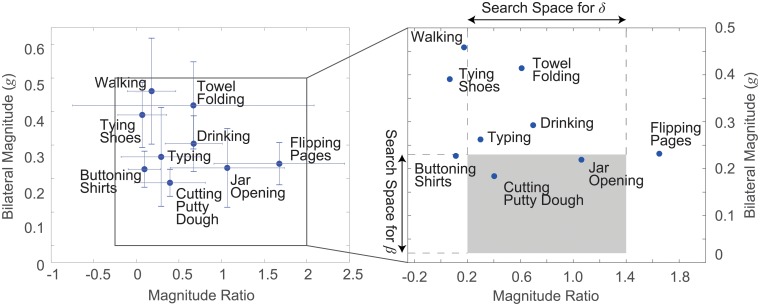
Scatter plots showing the relationship between the bilateral limb activity intensity |*a*_*r*_[*t*]| + |*a*_*r*_[*t*]| and the magnitude ratio *r*[*t*] for each motor task. (left) The scatter plot with the mean and standard deviation information computed across the participated subjects for each motor task. (right) The simplified scatter plot with only the mean information. The shaded area indicates the 2D search space used to choose the optimal *δ* and *β* values.

Fig 2 shows a scatter plot similar to plots used in previous studies [12, 17] to demonstrate the relationship between the intensity of bilateral limb activity (|*a*_*r*_[*t*]| + |*a*_*r*_[*t*]|) and the magnitude ratio (*r*[*t*]) estimated using the accelerometer data collected by means of the finger-worn accelerometers. The mean and standard deviation values of the two measures were computed for each motor task across all subjects participated in the in-laboratory experiments in order to simplify the visualization of the results. The search range to optimally choose the *δ* parameter value was set to be [0.2, 1.4] and the search used increments of 0.025. The search range to optimally choose the *β* parameter value was set to be [0.02*g*, 0.23*g*] and the search used increments of 0.01*g*. The shaded area in Fig 2 represents the 2D search space constructed based on the in-laboratory data.

### Parameter optimization for the proposed measure of upper-limb function using free-living experiment data

**Fig 3:**
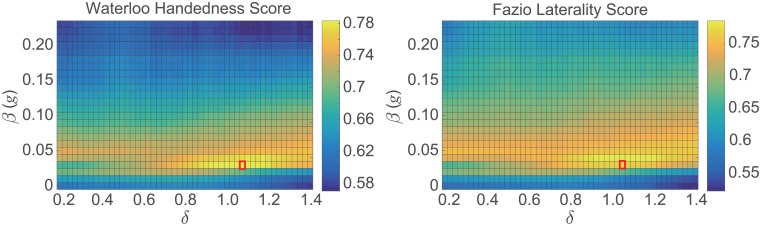
2D color map of the Pearson correlation coefficients between the proposed measure *M* and the Waterloo Handedness Questionnaire scores (left) and the Fazio Laterality Inventory scores (right) in the search space considered to determine optimal *δ* and *β* values. The red boxes at *δ* = 1.05 and *β* = 0.03*g* indicate where the maximum correlation coefficients were obtained for the two indices considered in the study.

Fig 3 shows a 2D color map representation of the Pearson correlation coefficients between the proposed measure *M* derived from the free-living experiment data and the Waterloo Handedness Questionnaire scores (left) and the Fazio Laterality Inventory scores (right) for the above-described search space used to determine optimal *δ* and *β* values. The maximum correlation coefficients for both indices were achieved with *δ* = 1.05 and *β* = 0.03*g*, which are indicated in red in Fig 3.

### Type and frequency of upper-limb use in free-living settings

Table 2 shows the percentage time of upper-limb use associated with different upper-limb activities (i.e., inactive vs. bilateral vs. unilateral) as obtained from data collected in a free-living environment using the above-mentioned optimal parameter values for *δ*, *β*, and the threshold used to determine active vs. inactive epochs. The mean and standard deviation values shown in Table 2 were computed by considering the data for all study participants. On average, subjects performed no upper-limb activities for 19 ± 14% of their time. The majority of the upper-limb activities involved both limbs and accounted for 53 ± 14% of the time, whereas only 28 ± 8% of the activities involved unilateral limb use.

**Table 2:** Mean and standard deviation of time percentage of upper limb activities.

Limb Activity	Time Perc. (%)
Inactive	19 ± 15
Bilateral Limb Use	53 ± 14
With Sufficient Intensity	42 ± 14
With Insufficient Intensity	11 ± 5
Unilateral Limb Use	28 ± 8
Right Limb with Sufficient Intensity	14 ± 6
Right Limb with Insufficient Intensity	3 ± 5
Left Limb with Sufficient Intensity	8 ± 4
Left Limb with Insufficient Intensity	2 ± 3

Unilateral limb activities (with sufficient intensity) that contributed to the proposed measure of real-world upper-limb function (i.e., *M* in(2)) accounted for approximately 22% of the overall data. In Table 2, the overall time percentage of right-limb use (with sufficient intensity) was substantially greater than that of left-limb use because the majority of study participants were right-handed (13 right-handed vs. 5 left-handed subjects). Table 3 shows the time percentage of unilateral limb use for the right-handed and the left-handed subjects separately (i.e., dominant vs. non-dominant limbs). The time percentage of dominant limb use for the right-handed and the left-handed subjects showed no statistically significant difference (*p* ≈ 0.14, *t*-test). Similarly, the time percentage of non-dominant limb use showed no significant difference (*p* ≈ 0.80, *t*-test) between the two groups. These results indicate similar dominant and non-dominant limb use patterns in real-world settings between right-handed and left-handed subjects. Vice versa, the percentage of dominant vs. non-dominant limb use across all study participants showed statistically significant differences (*p* < 0.01, paired *t*-test), thus indicating that subjects made substantially greater use of their dominant upper limb.

**Table 3:** Mean and standard deviation of time percentage of upper-limb activities involving sufficient intensity for right-handed and left-handed subjects.

Handedness	Limb Activity	Time Perc. (%)
Right-handed	Right Limb with Sufficient Intensity	17 ± 5
	Left Limb with Sufficient Intensity	7 ± 2
Left-handed	Right Limb with Sufficient Intensity	7 ± 4
	Left Limb with Sufficient Intensity	13 ± 6

**Fig 4:**
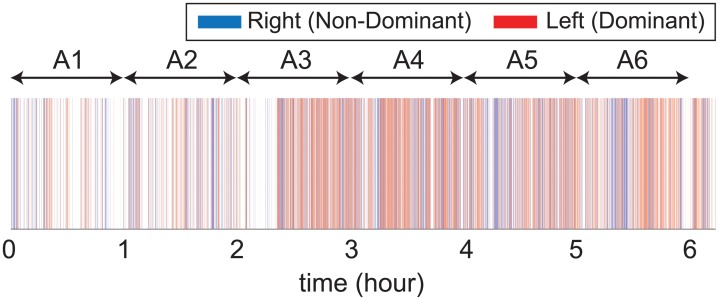
Illustration of unilateral use of the right (blue) and left (red) upper limbs for one of the study participants. Self-annotated activities (i.e., A1 to A6) for each hour of the monitoring period were as follows: A1—using the smart phone; A2—dressing and walking; A3—riding a moving vehicle, going to a coffee shop, using the smart phone; A4—going to a coffee shop and socializing; A5—walking, eating food, and using a smart phone; A6—writing, typing, and eating.

Fig 4 shows unilateral limb use data determined by the optimal values of *δ* and *β* in(2) for one study participant during the monitoring period (approximately 6 hours). The plot colors in blue the epochs associated with right-limb use and in red the epochs associated with left-limb use. Non-colored (white) time intervals indicate epochs involving insufficient-intensity unilateral limb use, bilateral limb use, or inactivity. The subject whose data is shown in Fig 4 was left-handed. The subject produced dominant (left) limb activity with sufficient intensity for 20.6% of the time and non-dominant (right) limb activity for 10.9% of the time. The subject produced insufficient-intensity unilateral activities for 4.3% of the time, bilateral limb activity for 55.6% of the time, and inactivity for 8.8% of the time. Activities that the subject self-annotated for each hour of the monitoring period are described in the caption of Fig 4. The plot shows that the subject predominately used the dominant limb. The intensity of use of the dominant limb was greater than the intensity of use of the non-dominant limb during the limb activity periods (e.g., A3—A6).

### Quantification of real-world upper-limb function

**Fig 5:**
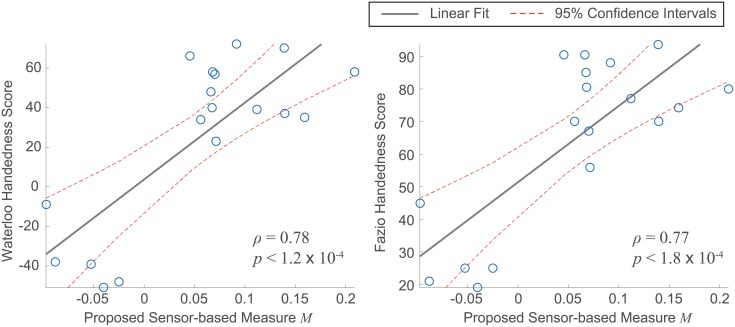
Scatter plots of the proposed sensor-based measure *M* and the two handedness indices utilized in the study. The sensor-based measure showed statistically significant agreement with both the Waterloo Handedness Questionnaire scores (left) and the Fazio Laterality Inventory scores (right). The grey solid line represents the linear fit, and the red dotted lines represent the 95% confidence intervals for the estimates.

Fig 5 shows scatter plots of the two handedness indices vs. the proposed sensor-based measure *M* of the study subjects, derived from the data collected in free-living conditions. The sensor-based measure showed statistically significant agreement with both the Waterloo Handedness Questionnaire scores (*p* < 0.01 with a Pearson’s correlation coefficient *ρ* = 0.78) and the Fazio Laterality Inventory scores (*p* < 0.01 with *ρ* = 0.77). These results establish the convergent validity of the proposed measure *M* with existing validated measures of real-world upper-limb function during the performance of ADLs.

Table 4 compares the proposed measure *M* to other sensor-based measures studied in the literature to date. As discussed above, we considered three different datasets to derive these measures: 1) finger-worn sensor data, 2) wrist-worn sensor data, and 3) a dataset obtained by computing the difference between the acceleration magnitude time-series derived from the finger-worn sensors and the wrist-worn sensors. Results in Table 4 show that 1) the proposed measure *M* outperformed existing measures of real-world upper-limb function and that 2) the results obtained using data collected by means of the finger-worn sensors are better correlated with the handedness indices than the results obtained using data collected by means of the wrist-worn sensors or data derived by combining the finger-worn sensor and the wrist-worn sensor data. In fact, the proposed measure based on the finger-worn sensor data produced the highest correlation with the handedness indices (i.e., *ρ* of 0.78 and 0.77 with the Waterloo Handedness Questionnaire scores and with the Fazio Laterality Inventory scores, respectively). The proposed measure based on the magnitude difference between the wrist-worn and finger-worn sensors also yielded high correlation with the handedness indices (i.e., *ρ* of 0.73 and 0.72 with the Waterloo Handedness Questionnaire scores and with the Fazio Laterality Inventory scores, respectively). Other than the proposed measure, there were two measures showing statistically significant correlations (*p* < 0.01) to the handedness indices: the median activity intensity ratio of the two limbs and the duration of sufficient-intensity dominant limb, both of which were derived from the finger-worn sensors.

**Table 4:** Comparison of the novel measure of upper-limb function *M* with previously investigated sensor-based measures.

Sensor-based Measurements of Upper Limb Use	Sensor Location	Pearson Coefficient (*ρ*)	
		Waterloo	Fazio
Median activity intensity ratio of the two limbs [17, 19]	Wrist	0.15	0.07
	Finger	0.61**	0.56**
	|Wrist-Finger|	0.36	0.40
Ratio of active use duration between the two limbs [20–22, 24, 46]	Wrist	0.19	0.13
	Finger	0.20	0.12
	|Wrist-Finger|	0.16	0.18
Ratio (between the two limbs) of the sum of the maximum acceleration magnitude within each one-second epoch [15]	Wrist	0.23	0.29
	Finger	-0.08	0.003
	|Wrist-Finger|	0.04	0.10
Percentage of the active dominant limb use with respect to the total monitoring duration [17, 21]	Wrist	0.32	0.38
	Finger	0.60**	0.59**
	|Wrist-Finger|	0.59**	0.55*
Median bilateral limb activity [17]	Wrist	-0.27	-0.30
	Finger	-0.11	-0.14
	|Wrist-Finger|	-0.08	-0.12
**Proposed measure** *M*: ratio of use duration between the two limbs	Wrist	0.45	0.45
	Finger	0.78**	0.77**
	|Wrist-Finger|	0.73**	0.72**

* *p* < 0.05,

** *p* < 0.01

In summary, results shown in Table 4 confirm our hypotheses that 1) finger-worn sensors allow one to derive a valid measure of fine hand movements and gross arm movements and 2) the index derived by comparing the time duration of limb use shows a strong agreement with self-reported real-world unilateral limb use indices.

## Discussion

This study examined the validity of a novel finger-worn accelerometer-based measure of upper-limb function and showed its suitability to monitor gross arm movements and fine hand movements. The results show that collecting data using finger-worn sensors and deriving the proposed measure of upper-limb use *M* leads to an accurate quantification of the amount of limb use during the performance of ADLs.

Fig 2 shows the face validity of the index derived from the finger-worn sensors. Higher mean acceleration magnitude values were observed when the data was collected during the performance of activities that required greater upper-limb activity intensity—such as walking, folding a towel, and tying shoelaces—compared to other activities tested in the laboratory. The results show that the finger-worn sensor can capture limb activities that involve both gross arm movements (i.e., walking and folding a towel) and fine hand movements (i.e., tying shoelaces), as we initially hypothesized. The mean magnitude ratio *r*[*t*] provided information regarding whether the two limbs were used together (e.g., walking, tying shoelaces, and buttoning a shirt) or a single limb was predominantly used (e.g., flipping magazine pages or opening a screw-top jar) to complete the motor tasks. Note that bilateral typing on a keyboard showed slightly greater involvement of the right limb as subjects often needed to press the Enter or Backspace keys. Folding a towel also mainly involved unilateral limb use as subjects often grabbed a corner of the towel and folded the towel using a single limb (while the other limb was used to stabilize the towel).

Convergent validity of the proposed measure of upper-limb function *M* was demonstrated based on its correlation with two well-established handedness indices (i.e., capturing self-reported upper-limb use preference during the performance of ADLs): *ρ* = 0.78 with the Waterloo Handedness Questionnaire scores and *ρ* = 0.77 with the Fazio Laterality Inventory scores as shown in Fig 5. The values of the two parameters utilized to derive *M*—i.e., *δ* and *β*—were empirically chosen to achieve maximum correlation with the handedness indices. The optimal values were identical for the Waterloo Handedness Questionnaire scores and the Fazio Laterality Inventory scores. It is worth noticing that a strong correlation has been also shown between these two handedness indices [39]. Hence, the fact that the same *δ* and *β* values led to maximum correlation with both handedness indices was not an unexpected result.

Categorizing upper-limb activities can provide clinically important information regarding how individuals use their limbs in real-world settings [9, 13, 14, 17, 22]. The values of *δ* and *β* used to derive the proposed measure *M* allowed us to categorize upper-limb activity into bilateral vs. unilateral activities. The average time distribution of upper-limb activities summarized in Table 2 is comparable with the results of a previous study by Kilbreath and Heard [30]. The authors conducted an observational study on the type and frequency of upper-limb activities performed by healthy older adults. The study reported that subjects spent 54 ± 10% of their time engaged in bilateral activities, 29 ± 10% in unilateral activities, and 17 ± 7% in no upper-limb activity. Our results showed a similar statistics: 53 ± 14% for bilateral activities, 28 ± 8% for unilateral activities, and 19 ± 14% for no upper-limb activities.

Table 3 shows that right-handed and left-handed subjects made greater use of their dominant limb during the performance of ADLs in free-living conditions. This finding concurs with previously reported results [52]. To the best of our knowledge, we have reported for the first time that wearable sensors can be used to derive information concerning limb use ratio according to the self-reported limb preference in healthy individuals. Previous studies based on wrist-worn accelerometers reported a roughly equal amount of activity of the dominant and non-dominant limbs [9, 13, 14, 22], which is a counter-intuitive finding in striking contrast with self-reports.

### Clinical applications

The proposed index *M* integrates limb activity intensity and ratio into a single measure by computing the ratio of unilateral activity duration of the two limbs. This measure similarly quantifies what the standardized clinical measure for real-world limb use (i.e., the MAL-AOU) aims to measure, and thus, has great potential for providing an accurate measure of limb use in individuals with hemiparesis (e.g., stroke survivors). We anticipate that the proposed sensor system will be most applicable to individuals with mild impairments and functional limitations of the stroke-affected upper limb with impairments and functional limitations primarily affecting the hand [53, 54]. The sensor system will allow clinicians to evaluate the impact in real-life conditions of the prescribed rehabilitation regimen as it will enable evaluating how patients improve their motor performance—in response to the intervention—for bilateral vs. unilateral tasks and fine hand movements vs. gross arm movements. This characteristic of the proposed system could enable individually-tailored, optimal therapeutic interventions [17, 55].

With its ability to continuously monitor upper-limb performance in a free-living environment, wearable technologies have great potential for providing clinicians with a tool suitable to maintain and possibly improve the motor skills recovered during the initial rehabilitation period by developing data-driven personalized interventions. For example, finger-worn sensors could combined with mobile devices (e.g., smart phones) to provide personalized feedback showing current progress and suggesting appropriate goals to promote high-dosage motor practice [56–58]. Despite the imperative need for such a system, the optimal configuration of the intervention components, the individual tailoring of the intervention goals, the design of optimal feedback components, the most suitable medium and timing to deliver feedback, and ways to share data among the stakeholders (patients and clinicians)—remains unknown and warrants future work.

### Limitations

One limitation of this study is that the proposed finger-worn sensor cannot explicitly detect and categorize upper-limb movements into 1) purposeful movements aimed to accomplish a goal-directed task vs. 2) non-purposeful movements that are the byproduct of movements of other body segments (e.g., arm swing activity during gait) or other sources of movements (e.g., acceleration of a moving vehicle when riding it). Categorization of upper-limb movements with respect to purpose can provide clinically meaningful information [12]. The proposed index *M* categorizes the majority of non-purposeful movements as bilateral movements because they often involve both limbs with similar intensity (e.g., arm swing during gait or during sit-to-stand transitions). Riding a moving vehicle may introduce a non-human-generated acceleration that is captured by the sensor data. Although the proposed index *M* does not specifically identify if a patient is riding in a moving vehicle, the index can still detect unilateral vs. bilateral limb activities in the vehicle, since 1) *r*[*t*] is capable of recognizing activity difference levels between the two limbs and 2) the non-human-generated acceleration from the moving vehicle—for instance—is not reflected in the measure because *M* compares duration of limb activity rather than intensity.

Another limitation of the study is the relatively small sample size. A total of 18 subjects participated in the in-laboratory experiments and another 18 subjects in the free-living experiments. Despite the small sample size, our results show strong evidence of face and convergent validity. The number of left-handed and right-handed subjects who participated in the free-living experiments was not balanced (i.e., 13 subjects were right-handed and 5 were left-handed). However, considering that only 10% of the general population is left-handed [59], we believe that we collected a sufficient sample of left-handed subjects. Furthermore, the results in Fig 5 show a high correlation between the proposed index of real-world limb use *M* and the handedness indices. It follows that the proposed index can enable the binary classification of left-handed vs. right-handed subjects with 100% accuracy by drawing a vertical line around *M* = 0. All together, we expect that the proposed technique will generalize to a larger population. Having demonstrated face and convergent validity in this work, future studies will focus on examining the proposed technology in larger populations of older adults (otherwise healthy) and patient populations (e.g. stroke and traumatic brain injury survivors).

## Conclusion

This study demonstrates the validity of a novel approach to assess real-world unilateral upper-limb activities using finger-worn accelerometers. The ability of the proposed technology to quantitatively compare the contributions of the upper limbs to the performance of ADLs has tremendous potential for allowing clinicians to evaluate the impact in real-life conditions of the rehabilitation interventions. This is of paramount interest in individuals with upper-limb hemiparesis. We envision that the system will be most applicable to individuals with mild upper-limb impairments and mild functional limitations, and will be used in the clinic in conjunction with other standardized clinical assessment tools to assess how impairments and functional limitations respond to clinical interventions on a subject-by-subject basis.
